# Tools to Guide Radiation Oncologists in the Management of DCIS

**DOI:** 10.3390/healthcare12070795

**Published:** 2024-04-06

**Authors:** Maria Cristina Leonardi, Maria Alessia Zerella, Matteo Lazzeroni, Nicola Fusco, Paolo Veronesi, Viviana Enrica Galimberti, Giovanni Corso, Samantha Dicuonzo, Damaris Patricia Rojas, Anna Morra, Marianna Alessandra Gerardi, Chiara Lorubbio, Mattia Zaffaroni, Maria Giulia Vincini, Roberto Orecchia, Barbara Alicja Jereczek-Fossa, Francesca Magnoni

**Affiliations:** 1 Division of Radiation Oncology, IEO, European Institute of Oncology IRCCS, 20141 Milan, Italy; cristina.leonardi@ieo.it (M.C.L.); samantha.dicuonzo@ieo.it (S.D.); damarispatricia.rojas@ieo.it (D.P.R.); anna.morra@ieo.it (A.M.); marianna.gerardi@ieo.it (M.A.G.); chiara.lorubbio@ieo.it (C.L.); mattia.zaffaroni@ieo.it (M.Z.); mariagiulia.vincini@ieo.it (M.G.V.); barbara.jereczek@ieo.it (B.A.J.-F.); 2 Division of Cancer Prevention and Genetics, IEO, European Institute of Oncology IRCCS, 20141 Milan, Italy; matteo.lazzeroni@ieo.it; 3 Department of Oncology and Hemato-Oncology, University of Milan, 20141 Milan, Italy; nicola.fusco@ieo.it (N.F.); paolo.veronesi@ieo.it (P.V.); giovanni.corso@ieo.it (G.C.); 4 Division of Pathology, IEO, European Institute of Oncology IRCCS, 20141 Milan, Italy; 5 Division of Breast Surgery, IEO, European Institute of Oncology IRCCS, 20141 Milan, Italy; viviana.galimberti@ieo.it (V.E.G.); francesca.magnoni@ieo.it (F.M.); 6 Scientific Directorate, IEO, European Institute of Oncology IRCCS, 20141 Milan, Italy; roberto.orecchia@ieo.it

**Keywords:** ductal carcinoma in situ, breast cancer, radiotherapy, local recurrence, management

## Abstract

Similar to invasive breast cancer, ductal carcinoma in situ is also going through a phase of changes not only from a technical but also a conceptual standpoint. From prescribing radiotherapy to everyone to personalized approaches, including radiotherapy omission, there is still a lack of a comprehensive framework to guide radiation oncologists in decision making. Many pieces of the puzzle are finding their place as high-quality data mature and are disseminated, but very often, the interpretation of risk factors and the perception of risk remain very highly subjective. Sharing the therapeutic choice with patients requires effective communication for an understanding of risks and benefits, facilitating an informed decision that does not increase anxiety and concerns about prognosis. The purpose of this narrative review is to summarize the current state of knowledge to highlight the tools available to radiation oncologists for managing DCIS, with an outlook on future developments.

## 1. Introduction

The available body of literature shows that the addition of radiotherapy (RT) to breast-conserving surgery (BCS) for ductal carcinoma in situ (DCIS) of the breast reduces the risk of local recurrence (LR) by approximately 50% at 10 years [1]. The beneficial effect on local control concerns all types of DCIS, regardless of tumour size, grade, margin width, and patient age. As a consequence, the guidelines to inform radiation oncologists (ROs) of the postoperative recommendations are cautious about the possibility of omitting RT, although it is recognized that the magnitude of the benefit varies according to the risk profile [2,3,4,5].

Attempts to identify the subsets of DCIS, for which the LR risk without RT is reasonably small, have been repeatedly made. In an international survey addressing the practice pattern of North American and European ROs in the early 2000s, grade 1–2 lesions with 10 mm margins resulted in respondents being divided evenly in their recommendations, with 47.7% favouring observation and 52.3% advocating for RT, with a similar number of participants suggesting the need for tamoxifen (45.5%). As the DCIS showed adverse features (grade 3, narrow margins), more ROs (87–99%) would recommend RT and tamoxifen [6].

The decision making about RT omission lies in the perception of both physicians and patients of a small advantage in local control [3,7], reinforced by the fact that breast cancer-specific survival (BCSS) does not seem to be affected.

To assess the LR risk, the immediately available information for ROs includes clinical–pathologic features (CPFs) such as patient age, grade, size, presence of necrosis, and surgical margins, in addition to biomolecular markers, namely hormonal receptors and Ki-67 and HER2 expression. All of them are easily accessible in clinical practice and can be evaluated one by one, at a basic level, or, in a more structured manner, using prognostic index or nomograms.

Along with the traditional factors, multigene assays have been raising high expectations for improving patients’ selection. They have a niche use in clinical practice and still need further refinements and confirmatory studies for a more extensive adoption.

The challenge for the decision making revolves around not only the omission of treatment but also around the modality of RT delivery, including de-escalation (boost omission, partial breast irradiation—PBI) or escalation (dose intensification) programs [8,9,10,11].

This narrative review aims to present the tools available to ROs to aid in decision making regarding the management and treatment of DCIS.

## 2. Lessons from the Past

The most commonly agreed-upon threshold for the omission of RT implies accepting the risk of total LRs of ≤10% and invasive LRs of ≤5% at 10 years, although it must be emphasized that what is acceptable as reasonably low risk is a matter of debate and need to be properly addressed with patients [8,12,13,14,15]. Different risk estimate cutoffs from <10% to <16% for recommending BCS alone were given by three different Ros [15]. 

Historical studies based the omission of RT on histological factors, such as tumour size, grade, and surgical margins. These parameters have proven to be unreliable in achieving the abovementioned LR risk threshold, both in meta-analyses of the randomized trials [1,16,17,18] and two prospective single-arm trials [19,20] started in the late nineties. The Eastern Cooperative Oncology Group (ECOG) 5194 trial phase II study [19] included either grade 1–2 DCIS, up to 2.5 cm (cohort 1), or grade 3 DCIS, up to 1 cm (cohort 2). In both cases, excision with 3 mm free margins was required. Thirty percent received tamoxifen. At 10 years, the overall LR rate was 12%, of which 6% was invasive, in cohort 1 and 20.5% (of which 11% was invasive) in cohort 2. The Dana–Farberg phase II study [20] dedicated to grade 1–2 DCIS with a maximum diameter of 2.5 cm and at least 1 cm free margins experienced a 10-year total LR rate of 15.6%, with 4.9% being invasive. 

The same criteria (3 mm free margins, size up to 2.5 cm, grade 1–2) were adopted by the RTOG 9804 randomized trial, comparing BCS alone versus BCS plus adjuvant RT [21]. Considering the local events in the BCS-alone arm at 10 years as a reference point, the trial results did not exceed the abovementioned threshold. In fact, the 10-year LR rate was 9.2%, of which 4.3% was invasive. Unlike the previous studies, tamoxifen was taken by the majority of the participants (62%) and it was associated, alongside RT, with a lower LR rate at multivariate analysis (MVA). However, at a 15-year follow-up, the rate of invasive LRs without RT increased at a faster pace to 9.5%. The authors speculated that, although LRs tend to increase over time, the risk associated with surgery alone might be sufficiently modest to be deemed acceptable for selected patients. 

Further refinements in patient selection include broadening the number of CPFs and integrating them with biomolecular markers. To assess the safety of the omission of RT in the low-risk DCIS, the ROMANCE randomized trial [22] integrated CPFs (age 50 years or older, margins ≥ 2 mm, size ≤ 2.5 cm, nuclear grade 1–2, and no necrosis) with biomolecular Luminal A-like features (positive hormonal receptors, HER2 negative, low Ki-67). High expectations also come from genomic assays, namely Oncotype DCIS and DCISionRT [13,23,24].

## 3. The Pattern of DCIS Progression

Most in situ LRs stem from residual DCIS after BCS for primary DCIS. They typically occur early, within 5 years of diagnosis [25,26], and show a clonal relatedness with the primary DCIS in up to 92% of the cases [27]. Progression from low- to high-grade lesions is uncommon [28]. In the Sloane Project, high-grade DCIS relapsed as such in 73% of the cases [25]. 

Conversely, invasive LRs tend to occur later, with the median time of reappearance depending on the grade of primary DCIS. In the Sloane Project, the median time for invasive LRs was 76 and 131 months from initially high-grade DCIS to low/intermediate-grade DCIS, respectively [25].

Invasive LRs can derive from residual DCIS, which takes a longer period to acquire the malignant evolution [29], or from initially undetected coexisting invasive ductal carcinoma (CDI) [30]. In both these cases, they can present a high correlation and concordance in gene expression with primary DCIS. High-grade DCIS are usually estrogen receptor-negative (ER) and have higher amounts of copy number variations (CNVs), point mutations, aneuploidy, and cancer-related gene alterations, such as MYC, CCND1, ERBB2, TP53, and PIK3CA [29,31,32,33]. A line of research focuses on the utility of prognostic gene expression markers to differentiate indolent DCIS from potentially progressive DCIS. Several studies have shown that the type of DCIS is predictive of the type of subsequent invasive cancer, with low-grade DCIS reappearing as low-risk invasive BC and high-grade DCIS as high-grade invasive BC [33,34,35]. In the Sloane Project, only 12% of the primary low-grade DCIS relapsed as a high-grade invasive disease [25].

On the other hand, invasive recurrence can arise de novo, without any clonal relatedness with the primary DCIS. The proportion of LRs unrelated to primary DCIS (which can be as high as 29%) [27] can partly explain the difficulty in identifying robust prognostic markers for LR. At a phenotype level, the grade of the invasive recurrence which did not match with that of the primary DCIS is heterogeneous, ranging from 8% in the Sloane Project to 47% of the cases in the EORTC trial 10853 [25,28]. 

Several theories have been developed to describe the progression of DCIS towards invasive tumours [31,32,33,36]. The direct lineage model included either the bottleneck model, which explains the high concordance in the molecular profile, or the multiclonal invasion model, which accounts for the similar level of molecular heterogeneity between DCIS and the coexisting invasive tumours. To explain a certain amount of molecular discordance, the independent lineage model speculates that DCIS and coexisting invasive tumours originate from different initiating cells. Another model is the convergent evolution which hypothesizes that a certain type of invasive BC could emerge from different genotypes of DCIS upon which the tumour microenvironment intervenes by selecting the same genomic alterations. Changes in the tumour microenvironment [31,35,37] in order to identify prognostic markers able to predict the risk of progression are a new field of research. Signs associated with invasive evolution include the increased thickness of the myoepithelium, different directionality of collagen fibres, higher E-cadherin expression, altered pattern of vasculature surrounding the DCIS-affected ducts, large number of fibroblasts, myeloid cells, lymphoid cells and large adipocytes, with crown-like structures in the extracellular matrix [38]. Dense stromal TILs seem to be associated with a higher recurrence risk [39], especially in the HER2-negative DCIS [40].

## 4. Prognostic Value of Clinical and Histopathological Factors

The prediction of LRs is currently based on CPFs (age, palpable presentation, size, grade, comedonecrosis, multifocality margins status, etc.) [41,42,43,44,45]. They may be subject to confounding factors, bias, and interobserver variations which can further complicate the interpretation of data [41,46]. In addition, invasive and non-invasive recurrence of DCIS do not share the same prognostic factors, and analysing LRs as a whole can be another confounding issue. 

The parameters that are the cornerstones to defining the local risk, namely grade, size, and margin status, showed some weaknesses in the prognostic value. In particular, the classification of the grade suffers from inconsistency due to various grading systems, interobserver variability, and the presence of heterogeneity within DCIS [41,47]. In the Sloane Project [25], the agreement on grade within the UK External Quality Assurance Scheme was moderate, with a kappa value of 0.55 for high-grade DCIS. The discordance among pathologists was especially seen in the assessment of the intermediate grade. 

The relationship between grade and risk of recurrence after surgical treatment is controversial [47]. Some studies showed a trend in high-grade DCIS towards progression into invasive tumours [19,41], while others did not [25,48]. In the Sloane Project [25], high-, intermediate- and low-grade DCIS showed comparable amounts of LRs (9.2%, 9.7%, and 9.8%, respectively) and invasive LRs (4.9%, 6.7%, and 6.7%, respectively). 

Regarding the size, the Early Breast Cancer Trialists Collaborative Group meta-analysis [1] found an association between the DCIS size and the 10-year risk of LR after BCS, following the algorithm of the larger the size, the higher the LR. Size cut-offs to categorize DCIS at low risk varied across the literature (<15 mm, ≤2mm–≤30 mm), with ≤25 mm being the most accepted threshold [1,2,21,49,50,51]. However, pathologic size can be difficult to measure and often considered an estimate, because it largely depends on the sampling completeness. In the nomogram developed by the Sloan Kettering Memorial Cancer Centre (SKMCC), the tumour size was replaced by the number of excisions as a surrogate [52].

The recommended width of negative margins varies across the studies [53]. In the Sloane Project [25], there was a statistically significant lower rate of recurrence in case of ≥2 mm clear margins, which is consistent with the 2016 SSO-ASTRO-ASCO guidelines [54] and St Gallen recommendations [55]. To perform PBI [56,57], ≥5 mm free margins are suggested by the DEGRO guidelines and St Gallen panellists. In the randomized BIG 3-07/TROG 07.01 trial, a radial surgical margin of less than 10 mm was considered as non-low risk and deserving of a boost dose [49]. A more detailed analysis revealed that wider margins were protective towards ipsilateral invasive recurrence of high-grade DCIS, regardless of RT [25]. In the study by Lei et al. [15], the three ROs involved in the LR risk estimates ranked surgical margins as the primary factor, followed in descending order by grade, comedonecrosis, tumour size, and patient age. 

Another histopathological feature that presents inconsistency in description is comedonecrosis, especially regarding the minimum amount required to gain such a label [58]. In a recent survey of 35 experienced American breast pathologists, more than one-third did not agree on any single cut-off.

The age factor should always be considered in the cost/benefit treatment evaluation [53], since it is an independent risk factor for LR [59,60,61]. The 10-year LR risk ranged from 11.2% to 31% in women aged 40 or less and 3–9% in those over 40 [62,63]. Young patients seem to be at greater risk for invasive rather than in situ recurrence [34,60,61,64]. This finding may explain some slight effect on BC mortality [48,65,66,67] in younger women and may boost the survival benefit of RT [65,68]. On the other hand, young age is predictive of lower responsiveness to RT, as confirmed by multiple studies and meta-analyses [1,17,34,59,66,69,70]. The lesser benefit of RT is once again seen in preventing invasive recurrence [66].

## 5. Tailoring Radiotherapy to Clinical and Histopathological Factors

CPFs are routinely used in the decision making regarding the modality of RT delivery individually [71]. Biomolecular markers and biosignature might help not only to omit RT but also to personalize RT, providing additional information that allows a de-escalation approach for less aggressive DCIS and escalation strategies for radioresistant lesions [72].

### 5.1. Partial Breast Irradiation

Partial breast irradiation (PBI) can be offered to selected DCIS with non-palpable presentation, tumour size ≤ 2.5 cm, low or intermediate grade, and free surgical margins of at least 3 mm. Such tumour profile, which meet the low-risk definition according to the RTOG 9804 trial [21], is currently supported by the NCCN guidelines [4] and was included in the suitability group of the American Society of Radiation Oncology (ASTRO) PBI guidelines [50] published in 2017. Data from the literature showed that in about two-thirds of patients the site of LR is consistent with that of the primary DCIS [20]. However, DCIS can spread along the branches of the ductal trees which may extend beyond the topographic division of the breast into quadrants [28]. 

The ASTRO Task Force has recently updated the guidelines for PBI to inform clinical practice based on high-quality evidence from the literature. The panellists underlined that some categories of BC such as DCIS were less represented in the PBI randomized trials. Out of the eight randomized studies included in the literature review, four recruited DCIS alongside invasive BC for a total of 1527 patients (768 in the PBI arm). The panel recognized that DCIS features, even the most common ones, such as size, grade, and margins, were not systematically reported, thereby affecting subgroup analyses and limiting the expansion of the risk factors for the decision making. As a consequence, the quality of evidence is only based on “Expert Opinion”. Two randomized phase III PBI trials showed no difference in local control by histology and gave additional information on the DCIS characteristics [73,74]. In the Group Européen de Curiethérapie/European Society for Radiotherapy and Oncology (GEC-ESTRO) trial [73] only low- and intermediate-risk DCIS (Van nuys Prognostic index = 8) with 5 mm clear surgical margins were eligible, while in the National Surgical Breast and Bowel Project B-39 trial, all the types of DCIS were allowed, including 28% grade 3 and 12% hormonal receptor-negative tumours [74]. A recent meta-analysis comparing PBI and WBRT by Chua et al. [9] including six studies highlighted the importance of DCIS selection. In fact, DCIS in the Suitable group fared significantly better than those in the Unsuitable group [odds ratio 2.69, 95% CI (1.56, 4.67)]. Heterogeneous selection criteria explained the mixed results on outcome from retrospective studies published in the literature, with 5-year LR ranging from 2.6% to 19% [75,76,77,78]. In the study by Leonardi et al. [76], expanding the selection criteria to include HER2 negative status and low Ki-67 proved to be successful in pinpointing the best candidates for PBI, resulting in a 10-year LR rate of 4%.

In the updated guidelines, the ASTRO Task Force members strongly agreed on recommending PBI for small grade 1–2 DCIS, since PBI might represent the fair balance between efficacy and tolerance, considering the relatively low increase in LR without adjuvant RT. Conversely, the panel strongly recommended against PBI in case of positive surgical margins, age less than 40, and BRCA1/2 mutation carriers. Regarding the use of PBI for high grade or tumour size comprised between 2.1 and 3 cm, the strength of recommendation was conditional, and PBI was felt to be somewhat inappropriate, especially when both the risk factors were present [79].

Two randomized phase III trials of whole breast RT (WBRT) and PBI for early-stage invasive BC included DCIS and showed no difference in local control by histology [73,74]. Looking at the characteristics of the DCIS, in the Group Européen de Curiethérapie/European Society for Radiotherapy and Oncology (GEC-ESTRO) trial [73] only low- and intermediate-risk DCIS (Van nuys Prognostic index = 8) with 5 mm clear surgical margins were eligible, while in the National Surgical Breast and Bowel Project B-39 Trial, all the types of DCIS were allowed, including 28% grade 3 and 12% hormonal receptor-negative tumours [74]. A recent meta-analysis comparing PBI and WBRT by Chua et al. [9] including 6 studies highlighted the importance of DCIS selection. In fact, DCIS in the Suitable group fared significantly better than those in the Unsuitable group [odds ratio 2.69, 95% CI (1.56, 4.67)]. Heterogeneous selection criteria explained the mixed results on outcome from retrospective studies published in the literature, with 5-year LR ranging from 2.6% to 19% [75,77,78,80]. In the study by Leonardi et al. [80], expanding the selection criteria to include HER2 negative status and low Ki-67 proved to be successful in pinpointing the best candidates for PBI, resulting in a 10-year LR rate of 4%.

### 5.2. Tumour Bed Boost

Until recently, the delivery of boost dose to the tumour bed has been controversial, with some reports showing benefits for young women and others only for positive surgical margins [81,82]. An international survey [7] showed a great heterogeneity for boost indications: about one-third of the physicians never delivered boost and the remaining two-thirds split over delivering it always or only in the presence of risk factors. 

The ASTRO guidelines for WBRT recommended the boost dose in case of patient aged 50 or younger, grade 3 and close (<2 mm), or positive margins [83]. 

The results of the randomized phase III trial, the BIG 3-07/TROG 07.01 [49] definitely established the beneficial effect of the 16 Gy boost in non-low-risk DCIS patients, with an absolute gain in local control of 4.4% at 5 years. The “non-low-risk” characteristics of DCIS were defined by having at least one of the following factors: young age (≤50 years), symptomatic or initially palpable tumours, size ≥1.5 cm, multifocality, intermediate or high nuclear grade, central necrosis, comedo histology, and surgical margins less than 10 mm. The downside of delivering a boost includes a higher fibrosis and cosmetic deterioration [49,84].

### 5.3. Postmastectomy Radiotherapy 

LR after mastectomy is an uncommon event, being in the range of 1–1.4% at 5 years and 2.6–3.2% at 10 years [16,64,85,86]. Therefore, postmastectomy RT (PMRT) is not routinely considered for DCIS. Since the majority of LRs after mastectomy are invasive, the decision making on PMRT is not straightforward [48]. Although there is no standard definition of margins in mastectomy specimens, the evidence of close/positive margin is the most important factor since the risk of recurrence increases more than three-fold compared to negative margins [87], especially when coupled with other risk predictors, such as high grade or young age [88,89]. In the study by Rashtian et al., high-grade DCIS with resection margins less than 2 mm resulted in a 5-year LR rate of 16% compared to 2% with wider margins (*p* = 0.035) [90]. Using the USC/Van Nuys Prognostic Index (VNPI) [91], Kelley et al. observed that patients operated on with mastectomy for DCIS achieving a score of 10–12 were more likely to develop LRs than those presenting a lower score (9.6% at 12 years versus 0%) [92].

LR after mastectomy is an uncommon event, being in the range of 1–1.4% at 5 years and 2.6–3.2% at 10 years [16,64,85,86]. Therefore, postmastectomy RT (PMRT) is not routinely considered for DCIS. Since the majority of LRs after mastectomy are invasive, the decision making on PMRT is not straightforward [48]. Although there is no standard definition of margins in mastectomy specimens, the evidence of close/positive margin is the most important factor since the risk of recurrence increases more than three-fold compared to negative margins [87], especially when coupled with other risk predictors, such as high grade or young age [88,89]. In the study by Rashtian et al., high-grade DCIS with resection margins less than 2 mm resulted in a 5-year LR rate of 16% compared to 2% with wider margins (*p* = 0.035) [90]. Using the USC/Van Nuys Prognostic Index (VNPI) [91], Kelley et al. observed that patients operated on with mastectomy for DCIS achieving a score of 10–12 were more likely to develop LRs than those presenting a lower score (9.6% at 12 years versus 0%) [92].

On the other hand, it is uncertain whether the delivery of PMRT can statistically significantly decrease the LR risk. The meta-analysis performed by Kim et al. [87] did not show such a benefit (Risk ratio 0.50; 95% CI = 0.06–4.08, *p* = 0.52, I ^2^ = 0%), but the quality of the analysed studies was low. Therefore, for PMRT, an individualized cost/benefit assessment, especially weighing up additional risk factors (positive/close margins, high-grade, multifocality, comedonecrosis, and age < 50 years), is recommended. 

### 5.4. Fractionation Schedules

Moderate hypofractionation (40–42·56 Gy in 15–16 fractions over 3 weeks) can be offered to all types of DCIS (strong consensus by the ESTRO Advisory Committee in Radiation Oncology Practice (ACROP) [93]. It is supported by the phase III trial BIG 3–07/TROG 07.01 dedicated to non-low-risk DCIS [49] and by other phase III trials which included both invasive and non-invasive tumours [94,95].

A pragmatic approach to applying ultra-hypofractionation (26 Gy/5 fractions) based on the low likelihood of radiobiological differences in dose fraction sensitivity of DCIS compared with invasive disease [96] is being pursued by the British ROs, while the ESTRO ACROP panellists are split over its use as either a standard of care or within clinical controlled trials [93].

### 5.5. Omission of Radiotherapy

DCIS lends itself to de-escalation strategies since RT does not improve BC mortality, which is reported to be relatively low (1.1% and 3.3% at 10 and 20 years, respectively) [11,16,48,97]. It cannot be excluded that the lack of survival benefit might be a matter of insufficient statistical power, given the relatively small benefit of RT. As pointed out by Goldberg et al. [98] in their editorial, the number of DCIS patients needed to prevent a BC death should be 370.

The survey by Mathelin et al. [7] showed that 73% of the physicians are in favour of some kind of therapy de-escalation (surgery or RT) for selected low-risk DCIS, especially for elderly women.

International guidelines, such as NICE [3] and NCCN [4], are open to the omission of RT in low-risk DCIS, especially in ER-positive tumours receiving endocrine therapy, provided that the decision is made in agreement between physicians and patients [71]. The 2019 St Gallen panellists voted in favour of both RT and endocrine omission for selected DCIS: low- or intermediate-grade, absence of comedonecrosis, age > 50 years, wide free surgical margins, and preferably exceeding 0.5 cm [57].

The shared decision making implies comprehensive information on the evolution of DCIS and the treatment options. A web-based survey [14] showed that DCIS patients were often confused about the prognosis and uncertain about the treatment, highlighting the importance of an effective communication [99]. Paradoxically, Byng et al. found that women were less worried about the risk of invasive local recurrence than oncologists [100]. To help physicians and patients make an informed choice, the clinical utility of online tools and genomic assays was confirmed in dedicated studies [101,102].

## 6. The Added Value of Biomolecular Factors

To improve the prediction capability, biomolecular markers such as ER and human epidermal growth factor receptor 2 (HER2) have been introduced, alongside attempts to stratify the risk according to molecular subtype classification [41,103,104]. The overexpression of HER2 is very common in DCIS, especially in tumours with unfavourable profiles and at a higher risk of recurrence, but the clinical significance is uncertain and it is not routinely evaluated [43,105,106,107]. The association of HER2 overexpression with recurrence was not consistently reported in the literature [108] and it appeared to be statistically significant only for in situ LRs [106,107], suggesting that HER2 is involved in the first phases of DCIS development rather than in the pathways of invasive progression. To further confirm this hypothesis, the administration of two doses of trastuzumab in the NSABP-B43 trial resulted in a 19% reduction in in situ LRs, although this was not statistically significant [109].

In addition, HER2 overexpression predicts a more effective response to RT for in situ LRs rather than for invasive ones [55,107,110]. 

Hormonal receptors status is one of the most important prognostic factors for LRs, especially when associated with the absence of HER2 expression [107,108,111]. Regarding the use of endocrine therapy, in the UK/ANZ and NSABP B-24 studies, adding tamoxifen reduced all BC events, in situ LRs, and contralateral BC for both the overall population and the subgroup not receiving RT, but not for the irradiated patients [5,112]. In the NSABP B-24 study [113], adding tamoxifen to RT was more effective in preventing all BC events, invasive LRs, and contralateral BC compared to RT alone. 

More recently, low-dose tamoxifen (5 mg at a day) for a limited period (3 years) proved to be beneficial in reducing LRs by 50% over an extended period (10-year follow-up), minimizing the possible side effects of the endocrine therapy [114]. Recognizing the beneficial effect of endocrine therapy, the NCCN task force [115] and ASCO/College of American Pathologists [116] recommended testing ER using immunohistochemical analysis for DCIS.

In addition, other markers related to proliferation, disruption in cell cycle regulation, cancer development, progression, and malignant transformation such as the expression of KI-67, p16 and p53, COX-2, Annexin A1, etc., have been tested in the prediction models and need to be formally validated [41,47,108,117]. In a case-control study in a cohort of patients treated with BCS alone, COX-2, HER2, and periductal fibrosis were associated with an increased risk of subsequent invasive LR [42]. The triple-positive profile (p16, COX-2, and KI-67) was significantly associated with a higher risk of invasive LR [118]. Ki-67, which is immediately available in clinical practice, measures the tumour cell proliferation and it is often associated with high-grade DCIS and comedonecrosis [117]. Its association with LR risk is controversial, mainly due to the low interobserver reproducibility and the variability of cut-offs, with possible implications for clinical utility [119]. The predictive value of KI-67 can be enhanced by the combination with other biomarkers, such as p16 and COX2+ [117]. Rakovitch et al. [120] showed that the HER2 positive/Ki-67 positive (≥10%) expression on MVA predicted a higher risk of in situ LRs compared to other molecular subtypes. In the study by Lazzeroni et al. [104], the incidence of LRs increased with the increase in Ki-67, with a possible cut-off of 14% for categorizing women as low- and high-risk. The same 14% cut-off made RT more effective in preventing LRs. Specifically, the RT effect was stronger with higher KI-67 levels. By grouping patients into molecular subtypes, Lazzeroni et al. [104] found that Luminal A DCIS did not benefit from RT. 

The development of a set of biomarkers suitable for risk stratification in clinical practice is the goal of many research and programs, like PreCancer Genome Atlas [35].

## 7. Decision Support Tools: Imaging Biomarkers

The accurate identification of the DCIS extent and the recognition of multicentricity or multifocality are of paramount importance for the management of DCIS, not only from the surgical but also from the radiation oncologist’s point of view. In fact, in the light of the growing trend towards treatment de-intensification, radiologic information can be used to complete the whole picture and guide ROs in considering the option of omitting radiotherapy or opting for PBI. The main presentation of DCIS is calcifications on mammography, which currently represents the conventional workup for DCIS and the most common imaging modality used for early detection [121]. However, mammographic determination of the extent of DCIS usually depends on the presence of calcifications, making mammography unable to identify most sites of DCIS. Moreover, this may lead to an underestimation of tumour size because the noncalcified invasive portions of the tumour are not detected [122].

In this context, Magnetic Resonance Imaging (MRI) demonstrated a higher sensitivity to DCIS compared to mammography [123], representing a useful imaging tool in the local staging of invasive cancer and may help to correctly identify the size and extent of DCIS [124]. Nevertheless, while MRI has been shown to identify mammographically occult invasive disease [125], DCIS detected on MRI is generally more likely to be higher in grade than mammographically detected DCIS [123], and in some cases, MRI may overestimate the actual lesion extent given the enhancement areas related to the plethora of proliferative changes occurring within DCIS [124].

In the prospective observational study carried out by the University of Bonn, Germany, almost half of the grade 3 DCIS were missed by mammography and spotted by MRI alone [123]. Additional neoplastic foci either in the ipsilateral or contralateral breast were found in up to 6.2% of the cases [126]. In the study conducted at the MD Anderson [126], 3.9% of patients developed invasive BC shortly after the treatment of DCIS, leaving open the question of whether they were new primary tumours or residual DCIS that progressed to invasive tumours. As previously said, MRI shows a great accuracy in identification of tumour extension which is generally seen to be larger than that in mammography [126], raising the question about the risk of overestimation [127]. The literature is not consistent in demonstrating that the use of MRI increases the rate of mastectomy, diminishes the probability of close/positive margins leading to fewer re-excisions in BC [128,129], and reduces LRs. In a large retrospective study on more than 2000 DCIS treated with BCS, Pilewskie et al. found no association between MRI and lower LRs rates, regardless of RT [130].

The most interesting line of investigation is the association of radiologic features with DCIS characteristics. This is an active field of research since it represents the radiologic backbone for any treatment de-escalation, including the active surveillance programs [131,132,133]. The most common MRI findings include non-mass enhancement with focal or linear patterns [126]. The association of the type of DCIS with qualitative MRI can be used to predict DCIS score or the risk of invasive tumours [126,129]. 

### 7.1. Quantitative Imaging Biomarkers

In this scenario, imaging biomarkers and radiomic features that are objectively and quantitatively measured may aid in the correct detection and characterization of DCIS. Regarding quantitative features from MRI scans, a systematic meta-analysis by Ding and Colleagues [134] showed that the apparent diffusion coefficient (ADC) values in diffusion-weighted imaging (DWI) for DCIS patients were significantly higher than in the invasive disease, confirming a reliable diagnostic value of ADC in differentiating invasive DC and DCIS. 

In addition, there are currently some radiomics-based biomarkers that suggest that MRI-based DCIS radiomics phenotypes may aid in the correct identification of the size and extent, and in the prediction of DCIS recurrence. Chou and Colleagues, in a 2017 study, tested the capability of computer-aided heterogeneity analysis in evaluating DCIS histologic grade and receptor status in 55 breast MRI imaged DCIS [135]. Their results showed how one heterogeneity metric, the surface–volume ratio, was significantly different between high nuclear grade and non-high nuclear grade DCIS, confirming the radiomics potentiality to provide non-invasive insights into diverse tumour biology, with potential implications for clinical management.

Regarding recurrence prediction, Kim et al. investigated whether the background parenchymal features at preoperative MRI of 215 women were associated with recurrence in patients with DCIS after breast conservation surgery [136]. The results of the study reported that a higher parenchymal signal enhancement ratio around the tumour at preoperative MRI and larger histologic tumour size were independent factors associated with worse recurrence-free survival. Similarly, Luo and et al. investigated the association of MRI imaging features and DCIS recurrence [137]. Their results from 415 women showed that higher functional tumour volume of lesion and signal enhancement ratio were significantly associated with the risk of developing a recurrence. These findings suggest that these quantitative preoperative MR imaging features may be useful in tailoring therapeutic approaches of DCIS ductal carcinoma in situ to match the risk of recurrence.

### 7.2. CADx

For BC, computer-aided detection (CAD) systems have been developed to assist radiology experts in detecting and diagnosing breast mass during breast imaging evaluation. Many CAD systems are designed for different breast imaging modalities such as mammography, breast ultrasound, breast MRI, breast tomosynthesis, PET CT, and Thermal imaging [138]. CAD has proven to be especially beneficial in mammography tests involving dense breast tissue since it is exceedingly sensitive and can detect even tiny abnormalities. In this regard, Malich et al. [139] demonstrate the clinical usefulness of their CADx system, developed for mammography, at classifying between suspicious mass and microcalcification.

Regarding DCIS, Vidya et al. [140] 6 demonstrated the high sensitivity of CADx depicting DCIS on screening mammograms by using biopsy proved lesion location as the reference standard. Their CADx identified DCIS in 91% of the lesions on screening mammograms obtained in the year of the diagnosis; in addition, considering the screening mammograms obtained before the year of diagnosis, it identified DCIS in 70% of the lesions.

The main limitation in the applicability of DL algorithms is the lack of information on how the algorithms actually work. Furthermore, any decision-making process in the clinical setting should be driven by combinations of appropriate data; thus, efforts to improve the transparency, explainability, and intelligibility of these DL algorithms are warranted [138].

## 8. Decision Support Tools: Traditional Prediction Models

Well-known CPFs (age, grade, size, margins, multifocality, comedonecrosis, palpable presentation, hormonal receptor status, receipt of endocrine therapy, and comorbidities) [51,99,141] have been included in several nomograms to predict the LR risk with and without WBRT. The clinical utility is controversial since the linear algorithms behind the nomograms singularly weighted the parameters and do not consider the molecular interdependency [142,143]. To increase the predictive accuracy, the CPFs are combined with biomarkers and genomic assay [144,145,146,147].

### 8.1. The Van Nuys Prognostic Index

The Van Nuys Prognostic Index (VPNI) is a historical tool to predict the probability of recurrence to inform the subsequent therapeutic approach [148]. The first version of the VNPI included a combination of high nuclear grade and comedonecrosis. Over time, to heighten the prognostic value, the VNPI incorporated other variables, such as size, margin width, and age, each of which was assigned a score from 1 to 3. The total score of up to 6 did not benefit from RT, the intermediate score of 7 to 9 carried a 20% LR risk and benefited from RT, while the high score of 10 to 12 predicted a LR risk of 50% and required mastectomy [149].

To improve the predictive performance, the VNPI was further refined in 2010 by modulating recommendations according to the score and the width of the surgical margin in a large series of subjects included in a prospective database [91]. The refined VNPI suggested the feasibility of excision alone for score seven and margins of ≥3 mm and called for mastectomy in the case of scores eight and nine with margins of <3 mm and <5 mm, respectively. With the same intention of ameliorating the prognostic value, Altintas et al. successfully integrated a proliferative biomarker, the Genomic Grading Index (GGI), into the VNPI [150]. 

Although the VNPI is simple to use and provides a simple solution, depending on the score, some shortcomings have undermined its diffusion and use [141], in particular, the development from retrospective monoinstitutional series, the inconclusive attempts to perform external validation [142,151,152,153,154,155], the lack of consideration for hormonal receptor status, and the use of endocrine therapy. In addition, the threshold of 20% for recurrence at 12 years as a determinant of adding RT is not widely accepted, since it might be considered too high. 

### 8.2. The Prognostic Score

The prognostic score developed by Smith et al. [156] ranging from 0 (low risk) to 6 (high risk), incorporated the well-known predictive CPFs namely age, tumour size, and grade, with and without comedonecrosis. By analysing more than 14,000 subjects in the SEER database, they found that the LR risk increased by 22% with every 1-point increase in the prognostic score. The prognostic score can inform the treatment, with BCS alone significantly associated with low-risk category. However, the SEER database did not collect variables that can independently modify the outcome; therefore, findings from the studies based on SEER analysis must be interpreted with caution. The prognostic score proposed by Smith et al. was also used to predict the survival benefit of RT after BCS on more than 30.000 patients included in the SEER Program of the National Cancer Institute in the period 1988–2007 [65]. Higher nuclear grade, younger age, and larger tumour size proved to be RT effect modifiers for BC mortality, resulting in higher survival benefit of RT as the prognostic index increased. 

### 8.3. The Memorial Sloan Kettering Cancer Centre Nomogram

A well-known nomogram was conducted at the MSKCC [52] on 1681 consecutive DCIS patients treated with BCS alone. The nomogram included 10 CPFs and treatment variables (use of endocrine therapy/RT, age, family history, mammographic detection, margins, number of excisions, grade, necrosis, and surgical year) to provide the risk estimate of recurrence at 5 and 10 years. The model is available online (https://nomograms.mskcc.org/breast/ductalcarcinomainsiturecurrencepage.aspx, accessed on 14 March 2024).

The discrimination power (how well the nomogram can differentiate between individuals with different a outcome) was moderate, being a c-index of 0.688 after bootstrap validation [52]. The external validation met conflicting results. Overall, the c-index was toward the lower end of the moderate range, between 0.63–and 0.69 [157,158,159,160,161]. The calibration (how well the prediction probability aligns with the observed outcome) was also variable. In the study by Collins et al. [159], there was a high correlation between the predicted and observed LRs of 0.98 at 5 years and 0.95 at 10 years, while YI et al. [161] found an imperfect calibration, leading to an overestimation of the risk in some patients. Also in a Spanish cohort, Oses et al. failed to accurately predict the LR risk [160]. Some major criticisms stem from the fact that the nomogram did not incorporate tumour size and biomarkers such as ER, PgR, and KI-67 [162,163]. 

## 9. Decision Support Tools: Biomolecular Prediction Models

Acknowledging the limitations of the CPFs in risk stratification, the novel field of research is focusing attention on the gene expression profile in order to identify genetic changes supporting tumour progression and invasive initiation [144]. The main ongoing trials dealing with genomic signatures in the DCIS setting are reported in Table 1. High expectations are placed not only on providing more reliable estimates of LR risk but also on predicting the response to RT, thus achieving a tailored approach [24]. 

### 9.1. The Oncotype DX DCIS Score

The Oncotype DX DCIS score was partly developed from the 21-gene Oncotype DX Recurrence Score in use for invasive tumours and includes five reference genes (ACTB, GAPDH, RPLPO, GUS, and TFRC) and seven genes relative to proliferation (KI-67, STK15, survivin, cyclin B1, MYBL2, PR, GSTM1). This multigene expression assay provides estimates of both in situ and invasive LRs at 10 years after BCS alone, regardless of tamoxifen and RT, and it was validated in the Ontario and in the highly selected ECOG E5194 [23] populations. The DCIS score is scaled from 0 to 100 and prespecified cut-offs are used to define the risk categories: <39 for the low, 39–55 for the intermediate, and >55 for the high risk. In the ECOG E5194 validation set, low, intermediate, and high scores corresponded to 10-year LR risk of 10.6% (3.7% invasive), 26.7% (12.3% invasive), and 25.9% (19.2% invasive), respectively [23]. In the Ontario validation set [164], the 10-year rates of LR for low, intermediate and high scores were 12.7% (8% invasive), 33% (20.9% invasive), and 27.8%, (15.5% invasive). In both the validation studies, the DCIS score remained an independent predictor for LR on MVA, although the hazard ratio of other CPFs was of higher magnitude compared to that of DCIS score [23,164]. Additional shortcomings included the lack of appreciable difference in risk between the intermediate- and high-score groups, with the intermediate-score group even displaying a higher absolute risk. Furthermore, the low-score group undergoing BCS alone still experienced a 10-year LR risk above 10%, a threshold widely accepted as triggering the need for RT [141,144,165,166]. To note, the 95% Confidence intervals (CI) of the DCIS score were wide, especially for the invasive LRs (95% CI, 1.34–9.62), resulting in 10-year LR risk which spanned from 5.1% to 27.8% for the intermediate-risk and from 1.8% to 7.7% for the low-risk groups [167].

To further refine the prediction value of the Oncotype DX DCIS score, the Ontario and ECOG E5194 populations were pooled together [145]. The combination of the DCIS score; and age at diagnosis, tumour size and year of diagnosis improved the accuracy of prediction and allowed a better risk stratification. In a study [146] comparing the three models of 10-year LR prediction, the CPFs +DCIS score showed a slightly higher discrimination power compared to either CPFs alone or combined with HER2 and ER (0.7025, 0.6879, and 0.6825, respectively). The refined DCIS score showed high concordance with the MSKCC Nomogram, except in the case of close surgical margins (<2 mm) where the multigene expression assay underestimated the LR risk. This finding underlined the importance of the margin width, especially when RT is not planned. 

The DCIS score does not predict the RT benefit, therefore, the choice of delivering RT or not is based on the assessment of the baseline risk with BCS alone. In the study on an Ontario cohort of patients irradiated and not, the RT benefit was proportional to the absolute LR risk: the absolute reduction with RT was 5.6% for low-risk DCIS score group and 12.8% for the high-risk DCIS score. In a subgroup of patients with favourable CPFs, those with high-risk DCIS score had the LR risk reduced from 19.6% to 11.9% by adding RT, while for those presenting low-risk DCIS score the risk reduction was from 10.1% to 6% [168]. Therefore, the gene expression assay added value to the CPFs-based risk stratification of patients receiving BCS alone. 

Since the use of tamoxifen in the ECOG study was limited (<30%), the algorithm for the DCIS score was designed to be unaffected by the use of endocrine therapy. Although in an exploratory analysis, the association of the DCIS score with LR risk was consistent with or without tamoxifen, the benefit of endocrine therapy [61,112] might lead to slightly lower overall BC events, altering the DCIS score prediction to some extent.

The assay is costly and its cost-effectiveness has been questioned. Dedicated studies showed that any strategy including the DCIS score failed to be cost-effective using a Markov model, unless they incorporated utility-sensitive analyses, taking into consideration patients’ preferences and concerns [141,169,170]. Several studies [102,171,172] demonstrated the impact of the DCIS score in reducing RT recommendations, by up to 29% in the Duchess study [102]. Interestingly, when weighing the impact of different variables on decision making, the DCIS score was rated as the most impactful, followed by pathologic features, patients’ preferences, age, and comorbidities [172]. Data from the National Cancer Database (NCDB) from 2010 to 2016 showed that the use of the DCIS score increased over time, especially for those with favourable features for whom a low-risk score significantly reduced RT recommendations [173]. Among patients, the assay also reduced anxiety and decisional conflict [14,169]. The Canadian study called Prospective Evaluation of Breast-Conserving Surgery Alone in Low-Risk Ductal Carcinoma in Situ (DCIS) (ELISA) (NCT04797299) is going to test whether low-risk patients defined according to CPFs and DCIS score <39 have <10% LR risk at 10 years with BCS alone. 

### 9.2. The DCISion Score

The DCISionRT score (PreludeDX, Laguna Hills, CA, USA), comprises seven cancer-related genes (HER2, Ki-67, COX2, SIAH2, FOXA1, and p16 expression) combined with four CPFs (age, tumour size, margin status, and palpability). The DCISionRT score was developed from a nonlinear algorithm using a machine learning technique to select biomarkers correlated with recurrence and progression. The nonlinear algorithm accounted for molecular and CPFs interactions so that the weight given to each variable was not independent of that of the others [13]. 

To note, the score included HER2 expression, which is not routinely measured for DCIS, and the positive triplet of P16, COX-2, and KI-67, whose association with invasive LRs was previously described by Kerlokinske et al. [118]. The DCISionRT score has both prognostic and predictive value, being able to discriminate patients at low and elevated LR risk and to assess the benefit of RT. The biological signature consists of a continuous risk score on a scale from 0 to 10. The 10-year absolute risks of total LRs and invasive LRs significantly increased with increasing DCISionRT score. For pragmatic reasons, a cut-off of three to categorize patients at Low (DS ≤ 3) and Elevated risk (DS > 3) was identified using the training datasets concerning the 10-year risk of ≤10% for total LRs (invasive and in situ) and ≤6% for invasive BC (including local and regional BCt events and distant metastases) [13]. Again, these thresholds can be a subject of discussion and need to be placed in the individual clinical context. The biosignature was cross-validated in four independent populations of retrospective series [13]. The difference in risk between irradiated and non-irradiated women in the Low-risk DS group was 1% (3% and 4% for invasive LRs and 7% and 8% for any ipsilateral LRs at 10 years, respectively). Conversely, in the Elevated-risk DS group such a difference reached 12% (9% and 15% for invasive LRs and 11% vs. 23% for any ipsilateral LRs between irradiated and non-irradiated subjects, respectively), resulting in a statistically significant RT benefit. A sizeable proportion of women (42%) considered at low risk based on CPFs (screen-detected, 2.5 cm size, G1–2, clear margins) was recategorized as Elevated-risk DS group, where the 10-year total LRs and invasive LRs risk with BCS alone would be of 23% and 31%, respectively. A substantial reclassification of the CPFs-based low-risk DCIS into the Elevated risk was also observed in other studies [174,175].

The clinical utility of the DCISionRT test was tested in the first part of the PREDICT study using a prospective multi-institutional registry [176]. Among 539 women, the biosignature brought about substantial modifications to RT recommendations between the pre-testing and post-testing contexts [177]. The post-test changes included either the annulment (46%) or the restoration (35%) of RT recommendations compared to the pre-test decision making. On the whole, the DCISionRT test reduced RT recommendation by 20%. Interestingly, in 46% of the patients with DCIS characteristics according to the RTOG 9804 study (G1–2, 2.5 cm, screen-detected, no close margins) who were candidates for observation, DCISionRT test recommended RT in 36% of the cases. The DCISionRT test weighed up as the most important driving factor in the decision making (odd ratio of OR 43.4), greatly surpassing all the other CPFs [177]. The DS was also validated in the SweDCIS randomized trial study population [174], where the test was predictive for RT benefit in reducing invasive LRs using a DS threshold above 2.8, while the *p*-value for interaction of RT effect with the cutoff of three was not statistically significant for either total or invasive LRs. A new version of the DS biosignature taking into consideration the EGFR/HER2/KRAS biomarkers expression with a prespecified algorithm was tested by Vicini et al. [178] to pinpoint patients who remained at higher risk after RT, the so-called residual risk subtype (RRt). The EGFR/HER2/KRAS pathway is known to be associated with tumour progression and drug resistance [179]. In a combined cohort of patients from three institutions [178], three categorical risk groups were defined: (1) Low-risk group (DS ≤ 2.8 without RRt), (2) Elevated-risk group (DS > 2.8 without RRt), and (3) RRt group (DS > 2.8 with RRt). DCIS in the RRt group showed a more aggressive profile (grade 3, large size, and HER2 overexpression) and higher LR risk without RT, with a 10-year rate of total LRs of 42.1% and invasive LRs of 18.5%. Despite the beneficial effect of RT, the LR incidence in the RRt remained significantly higher compared with the irradiated counterparts of the Elevated-risk group (total LRs 14.7% vs. 4.9% and invasive LRs 6.5% vs 3.1%, respectively) calling for a dose intensification or alternative approach. The Elevated-risk group presented a higher LR rate with BCS alone (20.6% total and 10.9% invasive LRs) and significantly benefited from RT (10-year absolute reduction of 15.7%). Conversely, the Low-risk group not only showed a low 10-year LR rate (total LRs, 5.1%; invasive LRs, 2.7%), but also the absolute difference between irradiated and non-irradiated patients was very small (0.8% for total and 0.6% for invasive LRs). Some limitations included [180] the retrospective nature of the analysis, the small number of events and patients, and the validation of the test in a prospective randomized trial. Studies on cost-effectiveness showed that the DCISionRT test would be cost-saving and more effective compared to decision making based on CPFs [181], especially if the cost of the test did not exceed a certain price [182]. 

## 10. Conclusions 

The decision making about the most appropriate local management of DCIS is challenging for both ROs and patients. A summary of the main key points guiding the RO in the treatment decision is given in Table 2, while Table 3 reports a clinical case as an example. The main purpose of RT is fulfilled given the efficacy in reducing both invasive and non-invasive LRs across all the risk groups, even if the impact on BC-specific survival seems not to be detrimental in the general population.

Following the principle of the minimum effective treatment, many attempts have been made to pinpoint a subgroup dubbed as low-risk, carrying a maximum of 10% overall and 5% invasive LRs risk at 10 years without RT. 

So far, ROs have relied on CPFs for the decision making. Size, margins, grade, and age are immediately available and easily accessible in clinical practice. Considered individually or incorporated into nomograms or prognostic scales, they have intrinsic limitations as described above. Nevertheless, the RTOG 9804 study showed that size, grade, and margins gained some validity in achieving the above-mentioned goal in the intermediate/long term, especially in women undergoing endocrine therapy. There is an increasing drive for ROs to go beyond the CPFs and to delve into the biology of the tumour. Information on hormonal receptor status is of utmost importance, since the benefit of endocrine therapy is increasingly recognized and side effects can be minimized by the administration of low doses. Additional biomarkers, such as HER2, and Ki-67, need more robust evidence. For the time being, their role in DCIS is exploratory and needs to be placed into a broader context to inform the management (for instance, PBI to small, low/intermediate-grade DCIS not overexpressing HER2 and omission of RT for luminal A-like DCIS with KI-67 < 10%). It is hoped that shortly they can contribute to guiding therapeutic choices more actively. High expectations reside in the use of multigene genomic assays, to correlate the phenotypic with the genotypic expression. Along with their value as prognosticators, the ability to predict the response to treatment might help ROs promote a risk-adapted RT. Considering the uncertainty associated with more traditional models, it is not surprising that the biosignature score has been rated as the most impactful factor for decision making in dedicated studies. The biosignatures are bound to be the real game-changer for DCIS, once they are consolidated and validated in prospective studies.

## Figures and Tables

**Table 1:** Summary of the ongoing clinical trials involving biomarkers or biosignature for DCIS.

Trial ID	Status	Title	Inclusion Criteria	Endpoint and Primary Outcome Measure	Estimated/Actual Primary Completion Date
NCT02492607	Active, recruiting	Management of Low-Risk Ductal Carcinoma in Situ (Low-risk DCIS): a Non-randomized, Multicenter, Non-inferiority Trial, Between Standard Therapy Approach Versus Active Surveillance (LORD)	Pure and low-grade DCIS ≥45 years old Any menopausal status Pure and low-grade DCIS	Endpoint: efficacy of active surveillance vs conventional treatment for low-risk DCIS Primary outcome measure: ipsilateral invasive breast tumour-free rate at 10 years	December 2029
NCT02926911	Active, not recruiting	Comparing an Operation to Monitoring, With or Without Endocrine Therapy (COMET) Trial For Low-Risk DCIS: A Phase III Prospective Randomized Trial	Unilateral, bilateral, unifocal, or multifocal DCIS without invasive breast cancer ≥40 years old ECOG 0 or 1 No contraindication for surgery ER (+) and/or PR (+) by IHC HER2 0, 1+ or 2+ by IHC	Endpoint: efficacy of active surveillance ± endocrine therapy vs conventional treatment for low-risk DCIS Primary outcome measure: ipsilateral invasive cancer rate at 2 years	July 2028
ISRCTN27544579	Active, not recruiting	Surgery versus Active Monitoring for LOw RISk Ductal Carcinoma in Situ (DCIS (LORIS))	≥46 years old Non-high-grade DCIS	Endpoint: whether patients with low-risk DCIS can safely avoid surgery and whether patients who do require surgery can be identified by pathological and radiological means Primary outcome measure: Ipsilateral invasive breast cancer-free survival rate at 5 years	March 2030
NCT04916808	Active, recruiting	The AUS-PREDICT Registry for DCIS Patients with DCISionRT Testing (AUS-PREDICT)	≥25 years old DCISionRT™ test Histologically confirmed DCIS in a single breast Eligible for breast-conserving treatment	Endpoint: utility of the DCISionRT™ test in the diagnosis and treatment of DCIS. Primary outcome measure: change in treatment recommendations after the test results	May 2024
NCT02872025	Active, recruiting	Testing the Ability of Immunotherapy to Alter the Tumor Immune MicroEnvionment (TIME) and Reduce or Eradicate High Risk DCIS	≥18 years old 2 or more high-risk DCIS features (grade II-III, palpable mass, negative hormone receptor-negative, Her2 positive, young age (≤45 years), lesion greater than 5 cm) Extensive DCIS and a small component of invasive disease History of tamoxifen and/or aromatase inhibitor ECOG 0 to 1	Endpoint: change in the immune microenvironment of high-risk DCIS after immunotherapy. Primary outcome measure: efficacy and safety of intralesional mRNA-2752 administration measured by the change in the MRI tumour size/volume/enhancement	March 2024
NCT04797299	Active, recruiting	Prospective Evaluation of Breast-Conserving Surgery Alone in Low-Risk Ductal Carcinoma in Situ Defined by a Molecular Expression Assay Combined With Clinico-Pathological Features (ELISA)	>45 years old DCIS without microinvasion Tumour size ≤ 2.5 cm BCS with clear resection margins ≥ 2 mm or no residual disease on re-excision Oncotype DX DCIS score with a predicted 10-year risk of LR ≤ 10%	Endpoint: whether the combination of clinicopathological factors and the use of the Oncotype DX DCIS score can avoid radiation Primary outcome measure: Ipsilateral local recurrence	March 2035
NCT06075953	Active, recruiting	DCIS: RECAST Trial -Ductal Carcinoma In Situ: Re-Evaluating Conditions for Active Surveillance Suitability as Treatment: a Breast Cancer Prevention Pilot Study	≥18 years old Previous diagnosis of HR+ DCIS (at least 50% ER or PR and 2+;) with or without microinvasion	Endpoint: whether active surveillance monitoring and hormonal therapy in DCIS patients can be an effective management of the disease Primary outcome measure: Fraction of patients remaining on active surveillance at 7 months compared to control	November 2028
NCT03878342	Active, recruiting	Radiotherapy Omission in Low Risk Ductal in Situ Carcinoma Breast (ROMANCE)	≥50 years old ECOG ≤ 2 Microcalcifications on pre-biopsy mammography, unifocal, ≤25 mm or opacity without microcalcifications and no clinical palpable tumour Absence of suspicious residual microcalcifications Breast-conserving surgical excision Non-invasive DCIS Free margins (≥2 mm), or free margins following re-excision Low or intermediate nuclear grade Absence of extensive necrosis (≤30% of the lumen diameter) Immunohistochemical characteristics of luminal A subtype: ER ≥ 10%, PR ≥ 20%, HER2 negative (0/1+) or 2+ not amplified, Ki67 < 15%	Endpoint: use biological markers to identify subgroups of patients who could safely avoid RT Primary outcome measure: 5-year cumulative incidence of in-breast cancer recurrences	November 2029

**Table 2:** Summary of the main key points of the study.

Key Points	
**Prognostic value of clinical and histopathological factors**	
	Some weaknesses in the robustness of the traditional CPFs for risk stratification: - Low to intermediate grade: Show a moderate agreement due to various grading systems, interobserver variability, and heterogeneity within DCIS. - Tumour size: Difficult to be measured with precision, often considered an estimate. - Surgical margins: Minimum margin clearance varies across the studies and guidelines.
	- The age factor should always be considered in the cost/benefit treatment evaluation.
**RT Treatment**	
	**PBI** ○ Suitable for low-risk DCIS patients (non-palpable presentation, tumour size ≤ 2–2.5 cm, low or intermediate grade, free-surgical margins of at least 3 mm). **Tumour bed boost** ○ Beneficial in non-low-risk DCIS patients (≤50 years, symptomatic palpable tumours, size ≥ 1.5 cm, multifocality, intermediate or high nuclear grade, central necrosis, comedo histology, surgical margins less than 10 mm).
	**Postmastectomy RT** ○ Not routinely considered for DCIS, requires an individualized cost/benefit assessment, weighing up some risk factors, such as positive/close margins, high grade, multifocality, comedonecrosis, and age < 50 years.
**Biomolecular factors**	
	Overexpression of *HER2:* Frequent in DCIS, uncertain clinical significance, not performed routinely. It is statistically significantly associated with in situ LR [120]; predicts a more effective response to RT for in situ LRs [120]. Hormonal receptor status (*ER+*): One of the most important prognostic factors for LRs [11,107,108]; endocrine therapy (tamoxifen) alone or in combination with RT reduces all BC events [113]. *Ki-67*: Usually associated with high-grade DCIS [118]; incidence of LRs increases with the increase in Ki-67 [119,120]. It is shown to be also a predictor of radiotherapy response [104].
**Decision support tools: imaging biomarkers**	
	Identification of the DCIS extent and the recognition of multicentricity or multifocality are of paramount importance for the management of DCIS. Imaging biomarkers and radiomic features may be useful in the characterization of DCIS and in the prediction of recurrence risk:
	- Surface–volume ratio was significantly different between high nuclear grade and non-high nuclear grade DCIS [135]; - Higher parenchymal signal enhancement ratio around the tumour at preoperative MRI and larger histologic tumour size were independent factors associated with worse recurrence-free survival after conservative surgery [136]; - Higher functional tumour volume of lesion and signal enhancement ratio were significantly associated with the risk of developing a recurrence [137]. Computer-aided detection (CAD) systems proven to be especially beneficial in mammography tests involving dense breast tissue [139].
**Decision support tools: traditional prediction models**	
	The Van Nuys Prognostic Index (VPNI) [148]—current application is limited. Main key features: - Built on retrospective monoinstitutional series; - External validation failed; - Does not consider endocrine therapy and hormonal receptor status; - Threshold for RT recommendation of 20% at 12 years deemed too high; - Refined with the addition of margin status width. The Memorial Sloan Kettering Cancer Centre Nomogram (MSKCC) [52] Main key features: - Includes 10 CPFs and treatment variables (e.g., the use of endocrine therapy/RT); - Available online; - Biomolecular markers and tumour size not included; - Externally validated in five studies with moderate discrimination power and variable calibration.
**Decision support tools: biomolecular prediction models**	
	The Oncotype DX DCIS Score [166,169]: Multigene expression assay (12 genes) for estimates of 10-year risk of any LR after BCS, regardless of tamoxifen and RT. Main key features: - No measure of predictive accuracy (discrimination and calibration); - Validated in highly selected population; - Failed to discriminate intermediate risk from high risk in a larger cohort [164]; - Margin status not considered; - Designed to be unaltered by endocrine therapy; - Costly; - Improved accuracy by incorporating age, size and year of diagnosis (Refined DS Score); - It was shown to be the most impactful factor in the decision making, reducing anxiety and decisional conflict. The DCISion score: Comprises seven cancer-related genes (among which HER2 and Ki-67), related with recurrence and progression, and four CPFs; discriminates patients at low and elevated LR risk and assesses the benefit of RT [177]. The higher the score, the higher the absolute risks of total LRs and invasive LRs [176,177,181,182]. Main key features: - Need to be validated in prospective randomized trial; - It is the most important driving factor in the decision making; - The novel version considers the EGFR/HER2/KRAS biomarker expression, identifying patients who remained at higher risk after RT.

**Table 3:** An example of clinical case which can have different treatment recommendations.

A Postmenopausal Woman Aged 52 Years, Undergoing Breast-Conserving Surgery for DCIS with the Following Features: Size 1.8 cm, Grade 2, 3 mm Negative Margins, no Comedonecrosis		
According to the risk factors, she may be candidate for: - Whole breast RT without boost according to ASTRO guidelines for Whole Breast RT [83] - Whole breast RT with boost according to BIG 3-07/TROG 07.01 study [49] - PBI according to ASTRO guidelines [50,79] - RT omission according to RTOG 9804 study [21]		
Decision-supporting tools:		
- Van Nuys redefined score 7 [91] ->	LR risk 16% at 12 years	
- Smith [156] 2/6 points ->	Moderate risk	
- MSK nomogram [52]	With endocrine therapy	4% at 5 y; 7% at 10 y
	Without endocrine therapy	9% at 5 y; 15% at 10 y
Additional parameters		
Positive estrogen receptor status	If endocrine therapy, RT omission or PBI may be a viable option [112]	
HER2 overexpression	Whole breast RT may be a viable option, RT is effective in reducing in situ recurrence [109]	
High Ki67 (≥14)	Whole breast RT may be a viable option, RT is effective in reducing both in situ and invasive recurrence [104]	
Highly needed additional tools		
- Biosignature and genomic tests which can be used to tip the scales either in favour of RT omission or RT recommendation [13,23]. Oncotype DX gives an estimate of LR risk. The DCISionRT (PreludeDx) may also provide information for RT intensification. In the setting of clinicopathologically low-risk DCIS, the DCISionRT reclassified 42% of patients into the Elevated-Risk Group [13], while 12-gene Oncotype DX reclassified about 10% of patients as a high-risk DS, which resulted in a 10-year risk of LR after BCS alone of more than 19% [168]. Biosignature and genomic tests may become greatly impactful in DCIS management once they are prospectively validated in randomized clinical trials.		

## Data Availability

The data that support the findings of this study are available from the corresponding author upon reasonable request.
